# Spilt Milk: An Unusual Cause of Bilateral Chylothorax

**DOI:** 10.1177/2324709615583877

**Published:** 2015-04-22

**Authors:** Sara Matani, J. Rush Pierce

**Affiliations:** 1 University of New Mexico School of Medicine, Albuquerque, NM, USA

**Keywords:** neck dissection, laryngectomy, thoracic duct, bilateral chylothorax

## Abstract

We report a case of bilateral chylothorax without evidence of chylous fistula in a 62-year-old man following total laryngectomy and bilateral selective neck dissection for laryngeal cancer. Chylous fistulae, a well-known complication of neck dissection, occurs following 1% to 2% of these surgeries. On rare occasions, the chyle leak may communicate with the pleural space, resulting in chylothorax. This is a rare but potentially life-threatening complication. Bilateral chylothorax following neck dissection is even rarer, with less than 25 cases reported in the literature. Early diagnosis is essential to prevent complications. Physicians should have a high index of suspicion, especially when the postoperative effusions do not respond to diuretics. Though no evidence-based treatment guidelines exist, expert opinion recommends conservative management as first-line therapy. Our patient was effectively treated by conservative management. We postulate a mechanism whereby bilateral chylothorax occurred in our patient without a chylous fistula.

## Introduction

Chylous fistulae, seen as milky drainage in the neck, is an infrequent but well-known complication of surgical neck dissection occurring in 1% to 2% of operated patients.^1^ On rare occasions, the chyle leak may communicate with the pleural space, resulting in chylothorax. Bilateral chylothorax following neck dissection is even rarer.^2^ In 1907, Stuart first reported bilateral chylothorax following neck dissection.^3^ Very few cases have been reported since. A systematic review of the literature done in 2012 listed only 23 such cases. The average age of patients was 53.8 years, and 70% were female.^2^ Laryngeal cancer was the primary tumor in only 3 cases. We report a case of bilateral chylothorax in a male patient occurring after total laryngectomy and bilateral selective neck dissection for laryngeal cancer who did not have a chylous fistula. This complication in our patient was successfully treated with conservative, nonoperative measures. We postulate a mechanism whereby bilateral chylothorax occurred in our patient without a chylous fistula.

## Case Presentation

A 62-year-old man with hypertension and diabetes developed hoarseness and was diagnosed with squamous cell cancer of the larynx by biopsy. Following radiation therapy, he underwent total laryngectomy and bilateral selective neck dissection, with a left pectoralis major pedicled myocutaneous flap reconstruction. During the operation, a lymphatic duct leak was identified; the duct was clamped and repaired. The area was then inspected and found to be free of chyle.

**Figure 1.**
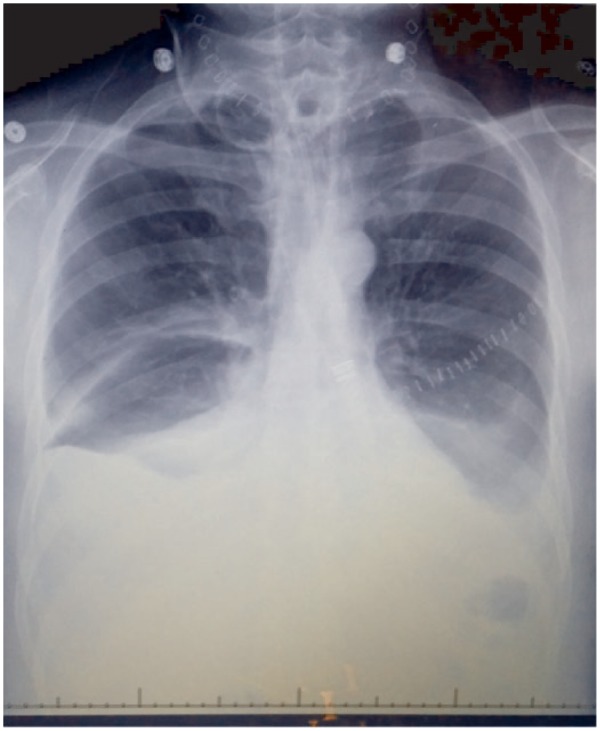
Chest X-ray 1 week after the surgery, interpreted as bilateral basilar atelectasis. Staples over the left thorax are a result of left pectoralis major pedicled myocutaneous flap reconstruction.

**Figure 2.**
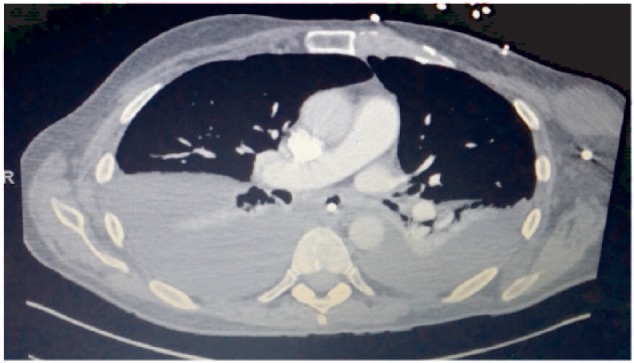
Computed tomography scan 1 week after the surgery—large right and moderate left pleural effusions.

Seven days after the surgery, the patient developed dyspnea and hypoxemia with an oxygen saturation by pulse oximetry of 82% on room air. Examination revealed dullness to percussion and decreased breath sounds at both lung bases. Chest X-ray was interpreted as showing bilateral basilar atelectasis (Figure 1). Computed tomography of the chest revealed bilateral pleural effusions (Figure 2).

**Figure 3.**
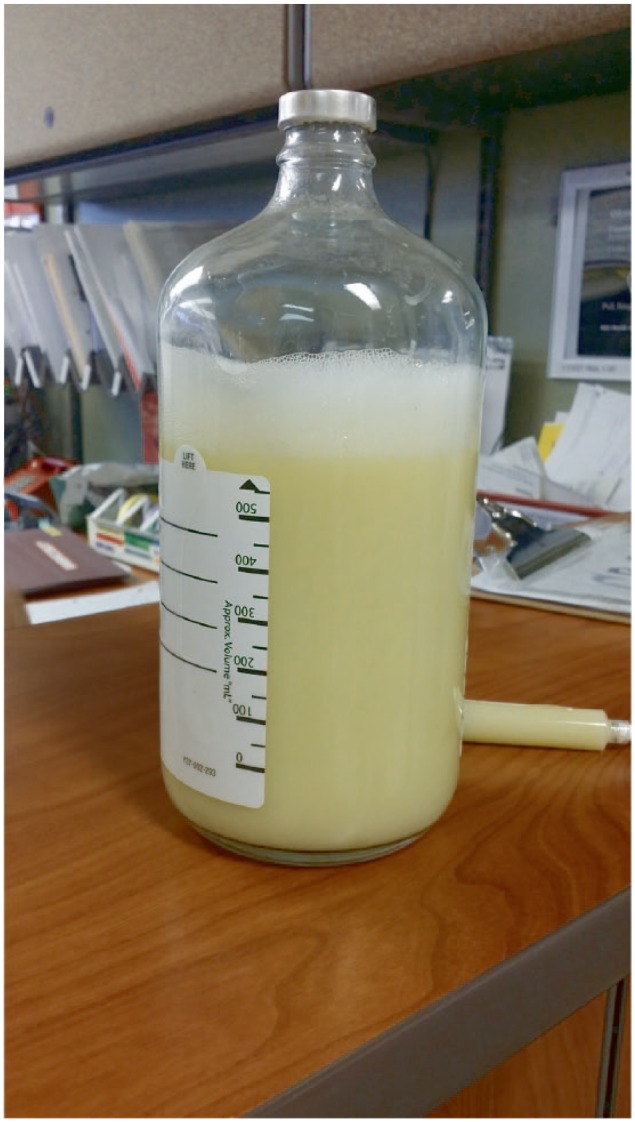
Thoracentesis—600 cc of milky fluid.

**Figure 4.**
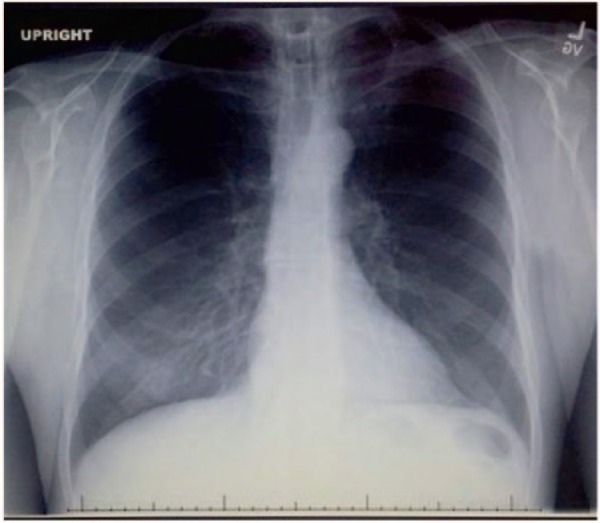
Chest X-ray 1 week after discharge—complete resolution of pleural effusions.

Thoracentesis of the right chest was performed and 600 cc of milky fluid was drained (Figure 3). Fluid analysis showed a transudative effusion with fluid triglyceride = 768 mg/dL. The next day ultrasonography confirmed the presence of bilateral chylothorax and a left-sided chest tube was inserted. No right-sided chest tube was placed at that time, due to the small size of effusion that remained after the previous day’s thoracentesis. Left chest tube drained 850 cc in the first 24 hours. The patient was begun on octreotide and placed on a low-fat enteral formula by Dobhoff tube that contained medium-chain fatty acids. Seven days later, his dyspnea had resolved, oxygen saturation was 95% on room air, and tube drainage was 40 cc/day. He was treated for 4 days with total parenteral nutrition (TPN), and tube drainage declined to 10 cc/day. Enteral feedings, TPN, and octreotide were discontinued, and he was given a regular diet. Two days later chest tube drainage was zero, the tube was removed, and the patient was discharged. Follow-up chest X-ray 1 week after discharge showed complete resolution of pleural effusions (Figure 4).

## Discussion

Lymph vessels from the peritoneal cavity and lower body come together below the diaphragm and give rise to the cisterna chyli, from which the thoracic duct originates. The thoracic duct passes behind the aorta through the aortic hiatus of the diaphragm and ascends the right side of the posterior mediastinum. At the level of the fifth thoracic vertebra, the thoracic duct passes to the left side, ascending behind the aortic arch and the left subclavian artery, and opening in the junction between the left subclavian vein and left jugular vein. This explains why injuries to the duct above and below the level of the fifth thoracic vertebra cause left-sided and right-sided chylothorax, respectively.^4^ When bilateral chylothoraces occur, damage of the duct is usually located where it passes the midline at the level of the fifth thoracic vertebra or as part of a more diffuse lymphatic condition.^5^

Autopsies have established that chlyothorax following neck dissection may occur without surgical perforation of the pleura due to extravasation of chyle under pressure into a closed or inadequately drained visceral compartment.^6^ If there is ligation or clamping of the thoracic duct in the neck, pressure builds up in the proximal portion of the duct and fluid may extravasate into the mediastinum. Then negative intrathoracic pressure during inspiration and the direct pleural maceration by chyle drives it into the pleural space.^7^ We believe this was the mechanism of formation of our patient’s bilateral chylous pleural effusions and occurred because his thoracic duct was clamped and repaired in the neck.

The thoracic duct transports 60% to 70% of ingested fat to the bloodstream; the usual concentration of fat in chyle is 0.4 to 6 g/dL. Chyle also contains proteins, white blood cells, electrolytes, coagulation factors, and large amounts of fluid.^4^ Thus, chylothorax may cause respiratory, metabolic, and immunologic derangements and can be life-threatening.^8^

Chylothorax should be suspected when pleural effusion occurs after neck dissection or when effusion fails to respond to diuresis. Analysis of pleural shows variable results, depending on the patient’s nutritional status. Generally fluid is exudative but can be transudative as in patients with amyloidosis, cirrhosis, heart failure, or nephrotic syndrome. Fluid is milky only in 50% of cases,^9^ and in other cases it may be serous, yellow, or bloody. Traditional cutoff values of triglycerides used to diagnose chylothorax may miss the diagnosis in fasting patients, particularly in the postoperative state. Triglyceride level of more than 110 mg/dL strongly supports the diagnosis (99% chance that the fluid is chyle). However, 15% of cases have triglycerides of less than 110 mg/dL, and 3% of cases with triglycerides less than 50 mg/dL.^10,11^ Diagnosis may be confirmed by the presence of chylomicrons in the fluid. This gold standard for diagnosis requires lipoprotein electrophoresis.^10,12^

Due to the paucity of published cases, evidence-based treatment guidelines are lacking.^8^ Among reported cases, nonoperative management is often successful. These conservative measures include chest tube drainage, low-fat TPN, medium-chain fatty acid diet, and octreotide. A medium-chain fatty acid diet is useful because these fatty acids are absorbed directly into the portal venous system rather than into the intestinal lymphatic system, thus reducing the amount of chyle produced. Octreotide is a long-acting somatostatin analog that acts directly on vascular somatostatin receptors to minimize lymphatic fluid excretion. In addition, octreotide increases splanchnic arteriolar resistance and decreases gastrointestinal blood flow, indirectly reducing lymphatic flow. Surgical management is usually employed when nonoperative strategies fail to resolve the effusion after 2 weeks, when severe metabolic complications occur, and when there is high outflow of more than 1 L/day for 7 days. Surgical procedures that have been used successfully include thoracic duct ligation, pleurodesis, and pleurectomy. The site of the leak may be located using lymphangiography or a fat meal mixed with methylene blue dye. Nonoperative interventional radiology techniques have also been used when conservative treatment fails. These include inserting a transjugular intrahepatic portosystemic shunt, percutaneous thoracic duct embolization, and percutaneous needle disruption of lymphatic pathways.^13^

Chylothorax is a rare but potentially life-threatening complication that may occur after neck dissection.^14^ This is usually due to surgical disruption of the thoracic duct with a resultant chylous fistula. Early diagnosis is essential. Our case illustrates that physicians should have a high index of suspicion, even in patients without a chylous fistula, and especially when postoperative effusions do not respond to diuretics.^15^ Though no evidence-based treatment guidelines exist, treatment with chest tube drainage, octreotide, and low-fat diet is usually successful.
